# Resolution of Acute Respiratory Distress Syndrome-Induced Takotsubo Cardiomyopathy with Venovenous Extracorporeal Membrane Oxygenation

**DOI:** 10.21470/1678-9741-2023-0117

**Published:** 2024-10-14

**Authors:** Ishaq J. Wadiwala, Pankaj Garg, Wesley L. Allen, Si M. Pham, Mathew Thomas

**Affiliations:** 1 Department of Cardiothoracic Surgery, Mayo Clinic, Jacksonville, Florida, US; 2 Division of Cardiothoracic Anesthesia, Mayo Clinic, Jacksonville, Florida, US

**Keywords:** Respiratory Distress Syndrome, Takotsubo Cardiomyopathy, Extracorporeal Membrane Oxygenation, Vascular diseases, Ventricular Function.

## Abstract

**Introduction:**

Takotsubo cardiomyopathy (TTCM) can occur in acute respiratory distress syndrome (ARDS) and a few cases in literature were reported to be associated with hemodynamic instability. All these patients were managed with venoarterial extracorporeal membrane oxygenation (VA-ECMO).

Case presentation: We present two patients with ARDS-induced TTCM who were managed successfully with venovenous ECMO (VV-ECMO).

**Conclusion:**

Ventricular function in both patients fully recovered three days after ECMO initiation, and they were subsequently weaned from ECMO once pulmonary function improved.

## INTRODUCTION

**Table:** 

Abbreviations, Acronyms & Symbols				
ABG	= Arterial blood gas		LVEF	= Left ventricular ejection fraction
aPTT	= Activated partial thromboplastin time		MR	= Mitral regurgitation
ARDS	= Acute respiratory distress syndrome		PaCO₂	= Partial pressure of carbon dioxide
BP	= Blood pressure		PaO₂	= Partial pressure of oxygen
CAD	= Coronary artery disease		PEEP	= Positive end-expiratory pressure
CT	= Computed tomography		RVSP	= Right ventricle systolic pressure
DVT	= Deep vein thrombosis		TEE	= Transesophageal echocardiogram
ECG	= Electrocardiogram		TR	= Tricuspid regurgitation
ECMO	= Extracorporeal membrane oxygenation		TTCM	= Takotsubo cardiomyopathy
FiO₂	= Fraction of inspired oxygen		VA-ECMO	= Venoarterial extracorporeal membrane oxygenation
ICU	= Intensive care unit		VV-ECMO	= Venovenous extracorporeal membrane oxygenation
LV	= Left ventricular			

Takotsubo cardiomyopathy (TTCM) is characterized by transient, reversible, severe systolic and diastolic left ventricular (LV) dysfunction with regional or global wall-motion abnormalities in the presence of normal coronaries. If not adequately managed, TTCM can lead to life-threatening arrhythmia, ventricular rupture, or even death^[1]^.

Since the influenza pandemic in 2009 and, more recently, the COVID-19 pandemic, acute respiratory distress syndrome (ARDS) has been increasingly reported to provoke TTCM^[2]^. The use of venovenous extracorporeal membrane oxygenation (VV-ECMO) in patients with severe hypoxia secondary to ARDS has shown good outcomes compared to the progressive escalation of mechanical ventilation and high fractional inspired oxygen^[3,4]^. Traditionally, a requirement for VV-ECMO was intact biventricular function as blood is only removed, oxygenated, and then re-instituted into the right atrium. Cardiac output is not mechanically augmented and remains reliant on myocardial function. In patients with ARDS-associated TTCM, who require ECMO for profound and refractory hypoxia, venoarterial extracorporeal membrane oxygenation (VA-ECMO) is the standard of care to bypass the dysfunctional heart chambers en route to providing systemic oxygenation through mechanically driven cardiac output. As a result, due to the presence of LV dysfunction in TTCM, all the case reports and series that have reported the use of ECMO for TTCM due to ARDS, to date, have instituted VA-ECMO^[5,6]^. However, compared to VV-ECMO, VA-ECMO is associated with higher complication rates and increased management requirements^[5]^. This report presents the successful management of two patients with hypoxia-induced TTCM secondary to ARDS using VV-ECMO.

## CASE REPORTS

### First Patient

A 68-year female had a past medical history significant for coronary artery disease (CAD) managed by drug-eluting stent in right coronary artery, hypertension, diabetes mellitus and underwent bilateral lung transplant in November 2020 for pulmonary fibrosis. Her immediate posttransplant recovery was complicated by prolonged ventilation, delayed wound healing and acute lung rejection type A1 requiring prolonged hospitalization. During follow-up, the patient had repeated episodes of lung transplant rejection type A1 due to unknown reasons, gastroesophageal reflux disease, one episode of sepsis due *to Pseudomonas and Escherichia coli* infections and acute kidney injury that required antibiotics. Her most recent admission in March 2022 was prompted by worsening dyspnea, without orthopnea, aspiration, or wheezing.

A chest computed tomography (CT) scan showed new ground-glass opacities throughout right and left upper lobe, suggestive of an acute inflammatory process. Bronchoscopy revealed white to yellow mucopurulent secretions involving multiple bronchopulmonary subsegments of bilateral lungs. However, bronchoalveolar lavage culture was negative. An echocardiogram performed at admission showed normal biventricular function, with a left ventricular ejection fraction (LVEF) of 65% and a right ventricle systolic pressure (RVSP) of 36 mmHg. All valves were functioning properly. Her esophagogram was normal. Donor-specific antibody testing was negative. Doppler ultrasound revealed a nonocclusive deep vein thrombosis (DVT) in the right internal jugular vein, an occlusive DVT in the right mid-femoral vein, and nonocclusive DVTs in the right common femoral, popliteal, and gastrocnemius veins. During hospitalization, chest X-rays showed worsening diffuse interstitial thickening and hazy airspace opacities, suggestive of pulmonary edema or infection.

Antibiotics were escalated and the patient had an initial improvement followed by gradual deterioration of respiratory status over next week, requiring high-flow nasal cannula at 40 L/min with inhaled nitric oxide (iNO) and finally requiring intubation with mechanical ventilation. The patient was initially managed with pressure-controlled ventilation, with fraction of inspired oxygen (FiO₂) at 80% and a positive end-expiratory pressure (PEEP) of 5 cm of H₂O. Over the next two days, PEEP increased to 10 cm of H₂O, with iNO of 20 ppm and FiO₂ increased to 100% without significant improvement. In arterial blood gas (ABG) analysis, partial pressure of oxygen (PaO₂) was 89.7 mmHg and partial pressure of carbon dioxide (PaCO₂) was 30.8 mmHg. The patient was initiated on epinephrine 0.02 µg/kg/min and vasopressin 0.04 U/hr for hypotension. Epinephrine was gradually escalated, and norepinephrine was also initiated. With vasopressors, her heart rate was 108/min, sinus rhythm and blood pressure (BP) were 116/74 mmHg, without signs of congestive heart failure, pulmonary edema, or cardiogenic shock.

**Fig. 1:**
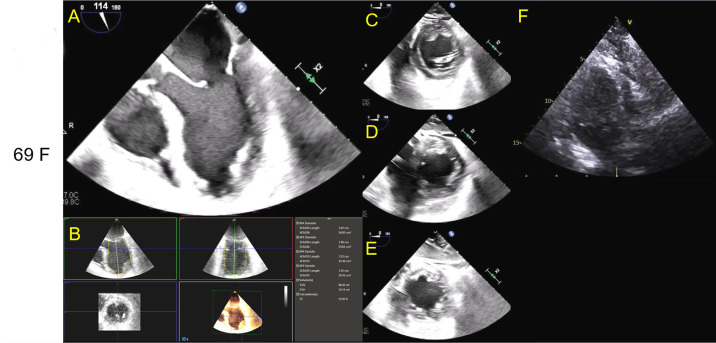
Intraoperative transesophageal echocardiography imaging of a mid-esophageal modified 5-chamber view (A), transgastric basal short-axis view, (C), transgastric mid-chamber short-axis view (D), and transgastric apical short-axis view (E). Left ventricular ejection fraction calculated at 14% via 3-D volumetrics (B). The images show basal contractility (C) with mid-chamber (D) and apical hypokinesis (E), consistent with Takotsubo/stress-induced cardiomyopathy. Post veno-venous extracorporeal membrane oxygenation on day 1 shows resolution of regional wall motion abnormalities in transthoracic apical 2-chamber view in Mayo Clinic format with the left ventricle on the left side of the screen (F).

High-sensitivity troponin T was 41 ng/L. Electrocardiogram (ECG) showed a normal sinus rhythm with tachycardia, a QRS duration of 96 ms, and diffuse ST-T elevation. Echocardiography revealed severe LV dysfunction with LVEF 14%, accompanied by layering LV thrombus and severe pulmonary hypertension (RVSP 68 mmHg) . LV apex was balloon out while only the basal segment was contracting, suggesting TTCM (Figures 1C, 1D, 1E). There was mild mitral regurgitation (MR) and moderate tricuspid regurgitation (TR). In view of deteriorating respiratory status, the multidisciplinary team decided to place the patient on VV-ECMO.

Link: Fig 1 A - https://youtu.be/VOpBL_X-Vc0

Link: Fig 1 B - https://youtu.be/74p87Ezq2iI

Link: Fig 1 D - https://youtu.be/hsV59zufHlA

Link: Fig 1 E - https://youtu.be/pnd5nHVZygA

Link: Fig 1 F - https://youtu.be/UYG_40C1iLQ

After administering a heparin dose of 100 u/kg, percutaneous cannulation of left internal jugular vein was performed with a 21 Fr arterial HLS cannula (Maquet Cardiopulmonary AG, Hirrlingen, Germany), and left femoral vein was cannulated with a 25 Fr venous HLS cannula (Maquet Cardiopulmonary AG, Hirrlingen, Germany). The cannulae were connected to ECMO circuit taking care to prevent air trapping. VV-ECMO was initiated at 3,000 rotations/min, with a flow of 3.2 L/min, sweep of 1.5 L/min and FiO₂ of 100%. Ventilation was reduced to rest vent setting with PEEP of 10 cm of H₂O, rate of 10 and pressure support of 10 cm of H₂O. To prevent the risk of bleeding, no further anticoagulation was initiated for the next 24 hours and the later patient was initiated on a bivalirudin infusion, aiming for an activated partial thromboplastin time (aPTT) of 40-60 seconds.

The patient tolerated the procedure well, with an immediate decrease in vasopressors after ECMO initiation. Within the next day, the patient was completely weaned off vasopressors and her pulmonary status improved. A follow-up echocardiography after 2 days showed normal biventricular function, with a LVEF of 65%, RVSP of 38 mmHg, mild MR, and mild TR (Figure 1F). The patient was successfully weaned off ECMO, decannulated after six days, and extubated after 12 days. During her hospitalization, her liver function tests and renal function remained normal, with good urine output. However, in the next four days, patient’s respiratory status deteriorated again, requiring intubation. The family decided to redirect the care to comfort, and the patient expired 30 days after hospitalization.

### Second Patient

A 78-year-old male, with a medical history significant for chronic renal insufficiency, secondary hyperparathyroidism, and Crohn's disease, who underwent proctocolectomy in 1997 with end ileostomy, underwent elective laparotomy, small bowel resection and revision of ileostomy in March 2022. Preoperative echocardiography showed normal biventricular function, LVEF of 62% and RVSP of 32 mmHg. All valves were functioning well. Coronary angiography showed minimal CAD.

The patient initially recovered well after surgery and was extubated and transitioning to oral intake on postoperative day 2. On postoperative day 6, the patient developed acute respiratory failure and septic shock due to aspiration, requiring intubation, antibiotic therapy, and vasopressors (vasopressin 0.02 U/hr). Liver function test was normal. Serum creatinine increased from 1.25 mg/dL to 1.83 mg/dL, with decrease in urine output, suggesting acute kidney injury.

**Fig. 2:**
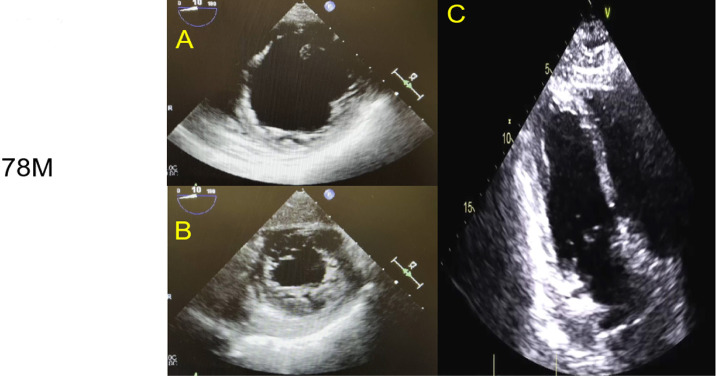
Intraoperative transesophageal echocardiography imaging of a transgastric mid-chamber short-axis view (A), and transgastric apical short-axis view (B). The images show severe mid-chamber akinesis (A) and apical hypokinesis (B), consistent with a reverse Takotsubo/stress-induced cardiomyopathy pattern. Post veno-venous extracorporeal membrane oxygenation on day 1 shows resolution of regional wall motion abnormalities in transthoracic modified apical 4-chamber view in Mayo Clinic format with the left ventricle on the left side of the screen (C) and a calculated 2-D biplane volumetric left ventricular ejection fraction of 59% per report.

Patient was initiated on continuous renal replacement therapy and initially managed with pressure-controlled ventilation with a FiO₂ at 70% and a PEEP of 5 cm of H₂O. Over the course of next 12 hours, patient developed refractory hypoxia despite positive pressure ventilation with FiO₂ of 100%, PEEP of 10 cm of H₂O and lung recruitment maneuvers. In ABG, PaO₂ was 46.2 mmHg and PaCO₂ was 66 mmHg. Contrast-enhanced chest CT was negative for pulmonary embolism and revealed multifocal pneumonia complicated by aspiration. Repeat echocardiography showed severe LV dysfunction, a LVEF of 30%, normal RV function, and normally functioning valves (Figures 2A and 2B). TTCM was diagnosed, and the patient was transferred to the intensive care unit (ICU) where a Swan-Ganz catheter was inserted. His cardiac output was 13 L/min and high-sensitivity troponin T was 37 ng/L. ECG showed normal sinus rhythm with tachycardia, QRS duration of 84 ms, and diffuse ST-T elevation. Over the next few hours, the patient’s condition deteriorated with hypotension, requiring multiple vasopressors (epinephrine 0.05 µg/kg/min, vasopressin 0.04 U/hr, norepinephrine 0.04 µg/kg/min and dobutamine 5 µg/kg/min), as well as high-flow nasal cannula at 40 L/min with iNO at 20 PPM. With vasopressors, heart rate was 104/min, sinus and BP was 122/76 mmHg. There were no signs of congestive heart failure, pulmonary edema, or cardiogenic shock. After multidisciplinary discussion, it was decided to initiate the VV-ECMO.

Link: Fig 2 A - https://youtu.be/vakrLdvCNFc

Link: Fig 2B - https://youtu.be/pOwaKVwdiz4

Link: Fig 2C - https://youtu.be/GMeYYhDixzs

Following the administration of 100 u/kg of heparin, percutaneous cannulation of the left internal jugular vein was performed with a 21 Fr arterial HLS cannula (Maquet Cardiopulmonary AG, Hirrlingen, Germany), and the left femoral vein was cannulated with a 25 Fr venous HLS cannula (Maquet Cardiopulmonary AG, Hirrlingen, Germany). The cannulae were connected to the ECMO circuit taking care to prevent air trapping. VV-ECMO was initiated at 2,800 rotations/min, with a flow of 3.0 L/min, sweep of 1.3 L/min and FiO₂ of 100%. Ventilation was reduced to rest vent setting with PEEP of 10 cm of H₂O, rate of 10 and pressure support of 10 cm of H₂O. To prevent the risk of bleeding, no anticoagulation was initiated for the next 24 hours, and later the patient was initiated on bivalirudin infusion aiming for an aPTT goal of 40-60 seconds. Intraoperative transesophageal echocardiography showed severe LV function with LVEF 15-20%, and normal functioning valve.

The procedure was uneventful, and the patient was shifted to the ICU on de-escalating dose of multiple vasopressors. In the ICU, repeat echocardiography after 24 hours showed normal LV size with mild basal-to-mid septal hypokinesis with LVEF of 59%. RV size and function were normal (Figure 2C). Vasopressors were weaned over the next 24 hours. Lung function improved over the next 2 days, and the patient was weaned off ECMO on postoperative day 2 and extubated on postoperative day 3. The ECG improved to normal. The patient gradually improved and was transferred to medicine department for further management and discharged on postoperative day 30. During hospitalization, the patient required continuous renal replacement therapy, followed by hemodialysis for 10 days, after which urine output improved. Echocardiography at the time of discharge showed normal biventricular function. At one-month follow-up, the patient was in good health.

## DISCUSSION

Takotsubo cardiomyopathy is completely reversible with resolution of the source and appropriate management of clinical findings. Etiopathogenesis of TTCM in patients with ARDS remains incompletely understood. In our cases, escalating inotropic and vasopressor requirement, ECG changes, mild elevation in troponin levels, and new-onset severe LV dysfunction of a non-vascular pattern after the onset of worsening hypoxia supported the diagnosis of ARDS-induced TTCM. Both patients developed progressively worsening hypoxia and became non-responsive to traditional therapies, prompting VV-ECMO institution. In combination to the cytokine storm and hyperinflammatory state seen in ARDS, profound hypoxia and its associated sympathetic surge most likely led to the development of TTCM^[2,7]^. Our diagnosis was further substantiated by the new-onset cardiovascular and hemodynamic signs that ensued early after the onset of worsening hypoxia and the rapid myocardial recovery following improved systemic oxygenation with the initiation of VV-ECMO. To our knowledge, no other reports exist in the literature detailing the use of VV-ECMO to manage ARDS-associated TTCM.

The decision to initiate VV-ECMO over VA-ECMO in our patients was made on the premise that severe hypoxia triggered TTCM, and that early correction would lead to TTCM reversal and recovery. There are significant patient and logistic benefits of VV-ECMO over VA-ECMO, such as lower anticoagulation requirements and bleeding complications, stroke risk reduction, lower nursing requirements, avoidance of arterial dissection/injury, and avoidance of elevated afterload on a severely depressed LV. Despite the depressed LV function on transesophageal echocardiogram (TEE), both patients were hemodynamically stable with adequate cardiac output on the current vasopressor/inotropic regimen without need for escalation. Our risk-benefit analysis did not find a significant advantage for mechanical circulatory augmentation above the critical need for systemic oxygenation. Veno-arterial-venous ECMO (VAV-ECMO) was considered a rescue plan if VV-ECMO alone did not result in the expected recovery.

Recovery of LV function in TTCM is highly variable and may take days to months^[8]^. In our patients, VV-ECMO initiation resulted in an immediate decrease in vasopressor requirements and myocardial function recovery over 48 hours. We believe that the institution of VV-ECMO improved myocardial and systemic oxygen delivery, significantly reducing the pathologic adrenergic surge and cytokine storm implicated in TTCM^[1,5,8]^. Rapid recovery of ventricular function in both patients shows that early diagnosis and treatment in hypoxia-induced TTCM can lead to accelerated ventricular recovery^[8]^.

## CONCLUSION

If diagnosed early, patients with ARDS-induced TTCM can be safely managed by VV-ECMO, keeping the option of converting to VAV-ECMO in case of deterioration.
